# 肺腺癌中免疫微环境特征与*EGFR*突变状态的相关性研究

**DOI:** 10.3779/j.issn.1009-3419.2023.101.07

**Published:** 2023-03-20

**Authors:** Hongyu ZHU, Peng CHEN, Guozhang DONG, Fanchen MENG, Zhijun XIA, Jing YOU, Xiangru KONG, Jintao WU, Fangwei YUAN, Xinyu YU, Qinhong SUN, Jinfu JI, Siwei WANG, Tongyan LIU, Lin XU

**Affiliations:** 210009 南京，南京医科大学附属肿瘤医院，江苏省肿瘤医院胸外科，江苏省肿瘤防治研究所，江苏省恶性肿瘤分子生物学及转化医学重点实验室; Department of Thoracic Surgery, the Affiliated Cancer Hospital of Nanjing Medical University, Jiangsu Cancer Hospital, Jiangsu Institute of Cancer Research, Jiangsu Key Laboratory of Molecular and Translation Cancer Research, Nanjing 210009, China

**Keywords:** 肺肿瘤, EGFR突变, 免疫微环境, 肿瘤异质性, 免疫治疗, Lung neoplasms, EGFR mutation, Immune microenvironment, Tumor heterogeneity, Immunotherapy

## Abstract

**背景与目的** 在全球范围内肺癌发病率和死亡率一直居高不下，而肺腺癌是其中最主要的一个组织亚型，表皮生长因子受体（epidermal growth factor receptor, EGFR）突变是肺腺癌的一个重要驱动基因突变。近年来以程序性死亡受体1（programmed cell death 1, PD-1）及程序性死亡配体1（programmed cell death ligand 1, PD-L1）抑制剂为代表的免疫检查点抑制剂（immune checkpoint inhibitors, ICIs）治疗在部分肺癌患者中疗效显著。有研究表明EGFR突变的患者对免疫治疗的获益程度有限，本研究旨在讨论肺腺癌患者中EGFR突变状态与各类免疫细胞浸润数量、空间分布的关系。**方法** 本研究纳入62例接受手术的肺腺癌患者。通过对手术切除的肿瘤组织进行不同区域多点采样，最终获取了223份肿瘤组织样本。通过基因检测获取了每份肿瘤组织的EGFR突变情况，包括突变丰度（variant allele frequency, VAF）以及突变亚型等信息。对肿瘤组织进行苏木精-伊红（hematoxylin-eosin, HE）染色、免疫组化染色、多重荧光免疫组化染色，并通过计算免疫组化评分获取各类免疫细胞在肿瘤组织中的浸润情况以及三级淋巴结构（tertiary lymphoid structure, TLS）的分布情况。**结果** 与野生型患者相比，EGFR突变肺腺癌患者肿瘤组织中CD68^+^巨噬细胞以及组织相容性复合物（major histocompatibility complex, MHC）II类抗原呈递细胞浸润更多，且MHC II类抗原呈递细胞空间分布的异质性较大，而CD56^+^自然杀伤细胞以及CD8^+ ^T细胞等浸润更低。EGFR VAF高的肿瘤组织与更低的CD3^+ ^T细胞、CD20^+ ^B细胞、CD56^+^自然杀伤细胞、CD68^+^巨噬细胞、CD8^+ ^T细胞等细胞浸润相关，其中只有CD3^+ ^T细胞分布的空间异质性较小。对于中国人群常见的两种EGFR突变亚型，EGFR外显子19缺失突变相较于EGFR外显子21 L858R突变肿瘤组织中有更低的免疫细胞浸润，但CD3^+ ^T细胞、CD56^+^自然杀伤细胞、CD68^+^巨噬细胞以及CD8^+ ^T细胞等免疫细胞分布的空间异质性更高。预后分析发现，CD3^+ ^T细胞、CD20^+ ^B细胞浸润程度高、TLS形成数量多以及CD8^+ ^T细胞高分布异质性的EGFR突变患者拥有更长的无病生存期。**结论** EGFR突变肺腺癌具有独特的低免疫细胞浸润的“非炎性”肿瘤微环境，不同突变亚型以及VAF的肿瘤微环境之间也表现出异质性，这些差异不仅体现在免疫细胞的浸润数量上，也体现在免疫细胞的空间分布情况。因此对EGFR突变肺腺癌的免疫微环境进行进一步更深入的研究对未来提高EGFR突变肺腺癌患者的免疫治疗疗效具有重大意义。

## Abstract (en)

**Background and objective** The morbidity and mortality rates of lung cancer remain high worldwide, and lung adenocarcinoma is one of the most important tissue subtypes of lung cancer. Epidermal growth factor receptor (EGFR) mutation is an important driver gene mutation for lung adenocarcinoma. In recent years, immune checkpoint inhibitors (ICIs), such as programmed cell death 1 (PD-1) and programmed cell death ligand 1 (PD-L1) inhibitors, have achieved remarkable efficacy in some lung cancer patients. Patients with EGFR mutations enjoyed limited benefits from immunotherapy according to recent studies. This study aimed to explore the relationship between EGFR mutation status and the spatial distribution as well as infiltration number of various immune cells in patients with EGFR mutant lung adenocarcinoma. **Methods** This study included 62 lung adenocarcinoma patients who underwent surgery. Through multi-point sampling of surgically removed tumor tissues in different areas, 223 tumor tissue samples were finally obtained. We aquired EGFR mutations status including variant allele frequency (VAF) and mutation subtype in each tumor tissue by genetic test. Afterwards, hematoxylin-eosin (HE) staining, immunohistochemical staining and multiplex fluorescence immunohistochemistry staining have been performed, therefore the infiltration of various immune cells and the distribution of tertiary lymphoid structure (TLS) in tumor tissues were obtained by calculating the immunohistochemical score. **Results** Compared with EGFR wild-type patients, patients with EGFR-mutant lung adenocarcinoma had more infiltration of CD68^+^ macrophages and major histocompatibility complex (MHC) class II antigen-presenting cells and higher spatial distribution heterogeneity of MHC class II antigen presenting cells in tumor tissues, while CD56^+^ natural killer cells and CD8^+^ T cells had lower infiltration. Tumor tissues with higher EGFR VAF were associated with lower cell infiltration such as CD3^+^ T cells, CD20^+^ B cells, CD56^+^ natural killer cells, CD68^+^ macrophages, CD8^+^ T cells, and only CD3^+^ T cells showed a lower spatial distribution heterogeneity. For the two common subtypes of EGFR mutations in Chinese population, tumor tissues with EGFR exon 19 deletion mutations have lower immune cell infiltration but higher spatial distribution heterogeneity of CD3^+^ T cells, CD56^+^ natural killer cells, CD68^+^ macrophages, and CD8^+^ T cells than that in EGFR exon 21 L858R mutant tumor tissues. Prognostic analysis found that patients with EGFR mutations with high degree of CD3^+^ T cells, CD20^+^ B cell infiltration and larger numbers of TLS formation and high spatial distribution heterogeneity of CD8^+ ^T cell had longer disease-free survival. **Conclusion** EGFR-mutated lung adenocarcinoma had a unique "non-inflammatory" tumor microenvironment with low infiltration of immune cells, and there was also heterogeneity in the tumor microenvironment among the tumors with different mutation subtypes and mutation abundance. These differences were not only reflected in the number but also the spatial distribution of immune cell infiltration. Hence, further studies on the immune microenvironment of EGFR-mutant lung adenocarcinoma were of great significance for improving the efficacy of immunotherapy in EGFR-mutant lung adenocarcinoma patients in the future.

在全球范围内，肺癌的发病率和死亡率一直居高不下，特别是死亡率位居所有癌症首位，而在我国肺癌的发病率与死亡率都位于榜首，根据中国国家癌症中心登记处2015年全国癌症统计数据，中国新诊断肺癌患者约73.33万人，中国肺癌患者死亡61.02万人（发病率35.92/10万；死亡率28.02/10万）^[1,2]^。根据组织类型肺癌主要分为小细胞肺癌（small cell lung cancer, SCLC）和非小细胞肺癌（non-small cell lung cancer, NSCLC），其中NSCLC约占肺癌的85%。NSCLC中最常见的组织学亚型是肺腺癌，长期以来治疗肺腺癌的手段以手术、放疗和化疗为主。EGFR突变是NSCLC的常见驱动突变，尤其是在亚洲人群中，发生率可达40%-60%，远高于西方人群（10%-15%）^[3,4]^。近年来逐渐兴起的表皮生长因子受体酪氨酸激酶抑制剂（epidermal growth factor receptor-tyrosine kinase inhibitors, EGFR-TKIs）治疗已经为EGFR突变肺腺癌患者的预后带来明显改善^[5]^，成为了EGFR突变肺腺癌患者的标准治疗手段。以程序性死亡受体1（programmed cell death 1, PD-1）及程序性死亡配体1（programmed cell death ligand 1, PD-L1）抑制剂为代表的免疫检查点抑制剂（immune checkpoint inhibitors, ICIs）逐渐在肺腺癌患者中展现出良好的疗效^[6]^，特别是对于PD-L1表达阳性的患者。在肺腺癌群体中的临床试验^[7,8]^结果表明，免疫治疗单药或者联合化疗的疗效都超过单纯化疗，然而有研究^[9,10]^表明免疫治疗对于EGFR突变的肺腺癌患者效果普遍不理想，免疫治疗在晚期EGFR突变患者中的应用仍有争议。EGFR突变丰度（variant allele frequency, VAF）是指在测序序列中EGFR的突变等位基因在所有等位基因（包括突变型和野生型）中的占比。发生在肿瘤进化早期的突变，通常被称为克隆突变或主干突变，一般比晚期的亚克隆突变具有更高的VAF，因此可以利用EGFR VAF值的高低作为评价EGFR早期克隆突变和晚期亚克隆突变的一种替代指标^[11]^。肺腺癌中EGFR突变包含了多种亚型，其中最常见的两种突变亚型为外显子19缺失突变和外显子21 L858R突变，占EGFR突变的90%以上。临床研究^[12⇓-14]^结果显示，免疫治疗对于EGFR外显子19缺失或L858R突变的患者的效果低于EGFR野生型NSCLC患者，且外显子19缺失组的疗效不如L858R突变组，而罕见EGFR突变的NSCLC患者相较于EGFR外显子19缺失或L858R突变的患者对ICIs有更好的反应，这可能与罕见EGFR突变的NSCLC患者肿瘤微环境（tumor microenvironment, TME）中PD-L1在CD8^+^肿瘤浸润淋巴细胞（tumor infiltrating lymphocytes, TILs）的高表达相关。不同EGFR突变的TME的异质性导致了对ICIs的不同免疫反应，因此进一步探索EGFR突变不同亚型的免疫学特征可能有助于我们选择受益于ICIs的人群。

多重免疫组化（multiplex immunohistochemistry, mIHC）技术可以通过多轮染色对一张切片实现同时检测多种标记物^[15]^，我们利用该技术对TME中的各类免疫细胞的分布特征以及浸润数量进行分析，描绘免疫微环境景观，并探讨肺腺癌中EGFR突变状态与免疫微环境特征的相关性。此外，我们通过对同一肿瘤组织使用四象限分割法进行多点采样，进一步探讨肿瘤组织内免疫细胞分布的空间异质性。

## 1 材料与方法

### 1.1 临床资料

本研究纳入了2017年4月-2019年1月确诊为肺腺癌的患者62例（表1）。所有患者在入组前均没有接受过新辅助治疗，并且术后病理都是被确认为肺腺癌。所有患者的组织学类型及肿瘤原发灶-淋巴结-转移（tumor-node-metastasis, TNM）分期均被两名经验丰富的病理科医生分别确认及复核，分期依据第八版美国癌症联合委员会 （American Joint Committee on Cancer, AJCC）分期系统。本研究获得南京医科大学附属肿瘤医院（江苏省肿瘤医院）伦理委员会批准，并且所有患者均填写知情同意书。经过严格质量控制，剔除少数肿瘤含量低、污染或者损坏的样本。对质控合格的肿瘤样本使用四象限分割法进行多点采样。肿瘤组织取样后进行石蜡包埋并制作组织芯片，以便后续进行免疫组化染色。

**表1:** 患者的临床特征

Category	n (%)
Age (yr)	
≥65	30 (48.4)
<65	32 (51.6)
Gender	
Male	35 (56.5)
Female	27 (43.5)
Smoking status	
Ever	50 (80.6)
Never	12 (19.4)
EGFR mutation	
Mutation	44 (71.0)
Wild type	18 (29.0)
Clinical stage	
I+II	29 (46.8)
III	33 (53.2)

EGFR: epidermal growth factor receptor.

### 1.2 二代测序

利用Illumina测序平台，对肿瘤样本进行高通量测序，获取超过400个肿瘤相关基因位点的突变情况。我们从测序结果中获得EGFR的突变状态，包括VAF及突变亚型等信息。

### 1.3 IHC

将石蜡切片进行烘片、脱蜡、水化，在3%双氧水中室温避光孵育10 min，蒸馏水冲洗，PBS浸泡5 min；加入一抗室温孵育2 h；PBS冲洗后加入二抗室温孵育30 min；滴加DAB显色剂，显微镜下观察5 min，苏木精复染后流水冲洗，封片观察。抗体信息：CD3（Dako A045229），CD20（Dako IS60430）。

### 1.4 mIHC

利用不同的抗体及荧光染料进行多轮免疫组化染色，抗体信息如下：CD56（Dako CD56 Clone 123C3, IS62830），CD68（Dako CD68, Clone PG-M1, IS61330），CD8（Dako CD8 clone C8/144B, IS62330），HLADR（Dako HLADR Clone TAL.1B5, M074601），PANCK（Dako CK Clone 34betaE12, M063001）。将石蜡组织进行4 μm切片。按照以下步骤进行：（1）脱蜡水合：①新鲜二甲苯浸片10 min，重复3次；②梯度乙醇浸片：100% 5 min；95% 5 min；70% 2 min；③灭菌水洗片1 min，重复3次；④10%中性福尔马林浸片10 min，灭菌水洗片1 min，重复3次。（2）微波修复抗原：①将脱蜡水合后的玻片置于修复杯中，用抗原修复液（1×）工作液浸没；②将修复杯置于微波炉内高火煮沸；③低火维持15 min；④取出后自然冷却至室温。（3）封闭：①去除玻片上残存洗液；②用组化笔圈出玻片上的样本区域，滴加封闭液，覆盖样本区域；③室温保湿震荡10 min。（4）一抗孵育：①去除玻片上的封闭液；②用移液器滴加稀释的一抗溶液，浸没样本区域；③室温保湿震荡孵育1 h；④用1×TBST Buffer浸洗玻片3 min，重复1次。（5）二抗孵育：①去除玻片上残存的洗液；②直接滴加HRP二抗工作液溶液，浸没样本区域；③室温保湿孵育10 min；④用1×TBST Buffer浸洗玻片3 min，重复1次。（6）荧光染色放大信号：①去除玻片上残存的洗液；②用移液器在玻片上滴加1×染料工作液100 μL（使用信号放大反应液按1:100稀释），浸没样本区域；③室温保湿震荡孵育10 min；④1×TBST Buffer浸洗玻片，室温浸片3 min，重复3次；⑤微波修复，室温下自然冷却；⑥灭菌水洗片1次，1×TBST Buffer浸片2 min；⑦本轮染色结束，追加后续染色。（7）封片：①去除玻片上残存洗液，滴加DAPI工作液，室温保湿孵育；②1×TBST Buffer浸洗玻片，室温浸片3 min；③用灭菌水洗片2 min；④待玻片微干，用移液器在玻片上滴加超强抗淬灭封片剂，浸没样本区域；⑤加盖玻片封片。（8）阅片：对染色后的组织片在荧光显微镜下观察并进行判读。

### 1.5 免疫组化评分

通过Halo软件计算免疫组化评分（H score），计算公式为：H-score=Σ（pi×i），式中pi表示阳性细胞数量占切片中所有细胞数量的百分数（0分-100分）；i代表染色强度，由弱至强为（0分-3分），最终评分范围为0分-300分。

### 1.6 统计学分析

本研究中的统计分析均使用R（Version 4.1.3）完成。对于两组以及多组之间连续变量的比较，使用Mann-Whitney U检验。对于空间异质性的评价，使用了R语言“Entropy”程序包计算Shannon entropy。为了比较病例的预后差异，使用Log-rank检验。所有P值均为双尾，P<0.05为差异具有统计学意义。

## 2 结果

### 2.1 EGFR突变与野生型的免疫细胞浸润分析

我们通过IHC和mIHC获得各类免疫细胞浸润情况，对免疫细胞浸润情况进行定量分析。我们发现EGFR突变型患者的肿瘤组织中有更少的CD56^+^自然杀伤细胞浸润（P=0.035），CD8^+ ^T细胞有更少的趋势（P=0.077），而CD68^+^巨噬细胞（P=0.005）以及组织相容性复合物（major histocompatibility complex, MHC）II类抗原呈递细胞浸润更多（P<0.001），此外EGFR突变患者肿瘤组织中PANCK标记的肿瘤细胞更少（P<0.001）（图1）。虽然EGFR突变患者肿瘤组织中抗原呈递细胞浸润更多，但是作为直接杀伤肿瘤细胞的CD56^+^自然杀伤细胞和CD8^+ ^T细胞浸润数量较少，特别是CD8^+ ^T细胞作为免疫治疗的效应细胞，其浸润数量的不足可能与EGFR突变患者免疫治疗的效果不理想具有潜在关联，而较高的肿瘤相关巨噬细胞浸润可能和肿瘤的进展、免疫逃逸有关。

**图1:**
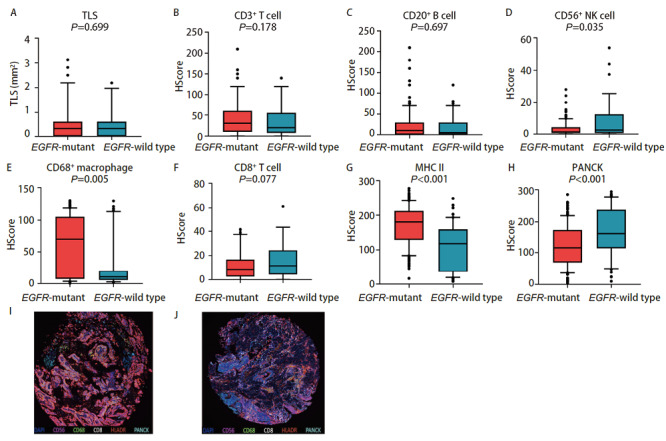
EGFR突变与野生型免疫浸润差异分析。 A-H：EGFR突变与野生型各类免疫细胞浸润以及TLS形成情况；I：典型的EGFR突变型肺腺癌mIHC图像（×100）；J：典型的EGFR野生型肺腺癌mIHC图像（×100）。

### 2.2 EGFR突变与野生型的免疫细胞分布的空间异质性分析

本研究通过多点采样的方式获取了同一患者肿瘤组织内部不同区域的组织样本，对不同区域的免疫细胞浸润情况进行分析，通过计算Shannon entropy来评价这些不同区域之间免疫细胞分布的空间异质性，以高Shannon entropy来表示高异质性。我们的分析结果显示，肿瘤组织内CD3^+ ^T细胞、CD20^+ ^B细胞、CD56^+^自然杀伤细胞、CD8^+ ^T细胞等免疫细胞分布的空间差异在EGFR突变型与野生型之间并不显著，只有MHC II类抗原呈递细胞在EGFR突变型的肿瘤组织内表现出较高的空间分布异质性（P=0.013）（图2）。

**图2:**
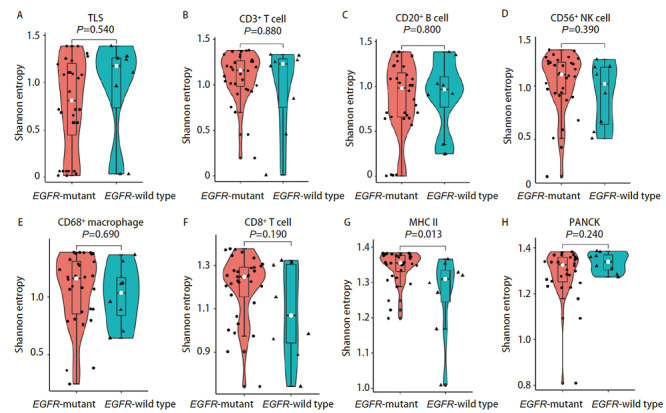
EGFR突变与野生型的免疫细胞分布的空间异质性分析。 A-H：EGFR突变与野生型各类免疫细胞浸润以及TLS形成的空间分布差异。

### 2.3 EGFR VAF与免疫细胞浸润分析

为了进一步分析EGFR突变状态与免疫微环境的相关性，我们将EGFR突变的肺腺癌样本以中位EGFR VAF值分为EGFR VAF高和EGFR VAF低两组。我们发现EGFR VAF高的肺腺癌肿瘤组织中，包括CD3^+ ^T细胞（P=0.014）、CD20^+ ^B细胞（P<0.001）、CD56^+^自然杀伤细胞（P=0.053）、CD68^+^巨噬细胞（P=0.049）、CD8^+ ^T细胞（P=0.001）等在内的免疫细胞浸润数量都是减少的，而且三级淋巴结构（tertiary lymphoid structure, TLS）形成的数量更少（P=0.002），此外PANCK标记的肿瘤细胞含量也更多（P<0.001）（图3）。有研究^[15]^表明EGFR VAF高的肺腺癌患者可以从EGFR-TKIs治疗中获益更多，我们发现EGFR VAF高的肺腺癌肿瘤组织中免疫细胞总体上浸润更少，肿瘤细胞含量多，而EGFR VAF低的肿瘤组织有着更高的免疫细胞浸润，也是表明了EGFR VAF低的患者如果不能从EGFR-TKIs治疗中获益，或许可以从免疫治疗中寻求更多获益。

**图3:**
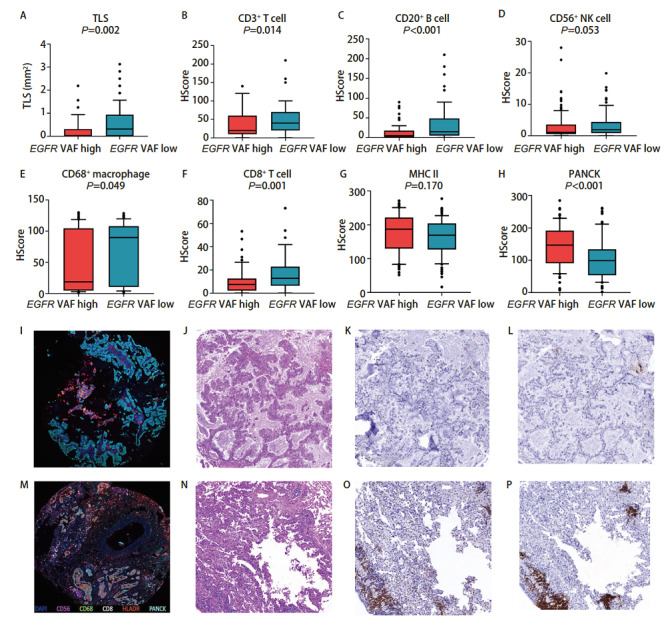
EGFR突变丰度高低的免疫浸润差异分析。 A-H：EGFR突变丰度高与丰度低的肺腺癌组织中各类免疫细胞浸润以及TLS形成情况；I-L：典型的EGFR突变丰度高的肺腺癌mIHC图像、HE染色图像以及IHC CD3和IHC CD20图像（×100）；M-P：典型的EGFR突变丰度低的肺腺癌mIHC图像、HE染色图像以及IHC CD3和IHC CD20图像（×100）。

### 2.4 EGFR

VAF与免疫细胞分布的空间异质性分析 我们的分析结果显示，多种免疫细胞在EGFR VAF高的肿瘤组织中都呈现出相对较少的趋势。接下来我们试图探究不同EGFR VAF下肿瘤组织内免疫细胞的空间分布异质性情况，结果表明，在EGFR VAF低与EGFR VAF高两组之间，TLS、CD20^+^B细胞、CD56^+^自然杀伤细胞、CD8^+ ^T细胞、抗原呈递细胞等免疫细胞分布的空间差异并不显著，只有CD3^+ ^T细胞在EGFR VAF低组表现出更高的空间异质性（P=0.046），CD68^+^巨噬细胞在EGFR VAF低组有空间异质性更高的趋势（P=0.079），但无统计学差异（图4）。

**图4:**
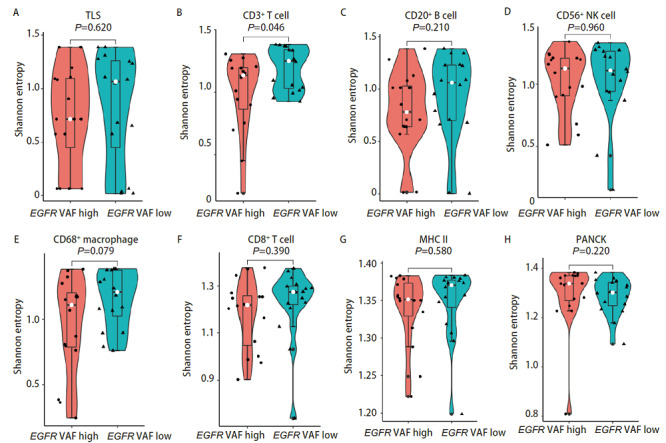
EGFR突变丰度高低的免疫细胞分布的空间异质性分析。 A-H：EGFR突变丰度高与丰度低的各类免疫细胞浸润以及TLS形成的空间分布差异。

### 2.5 EGFR常见敏感突变亚型的免疫细胞浸润分析

EGFR突变包含多种突变亚型，其中EGFR外显子19缺失突变与EGFR外显子21 L858R突变是最常见的两种突变亚型，两者都是EGFR-TKIs治疗的敏感突变，但是部分研究^[12]^显示两者对EGFR-TKIs以及免疫治疗有着不同的治疗反应，因此，我们对两者的免疫微环境特征进行分析。我们发现EGFR外显子21 L858R突变与外显子19缺失突变相比，其肿瘤组织中有着更高的CD3^+ ^T细胞（P=0.016）、CD20^+ ^B细胞（P=0.077）、CD68^+^巨噬细胞（P=0.008）、CD8^+ ^T细胞（P=0.032）浸润，而且TLS形成的数量也更多（P=0.005）（图5）。整体上EGFR外显子21 L858R突变肿瘤组织中各类免疫细胞浸润数量更多，特别是CD8^+ ^T细胞的较高浸润可能与更好的免疫治疗反应相关。

**图5:**
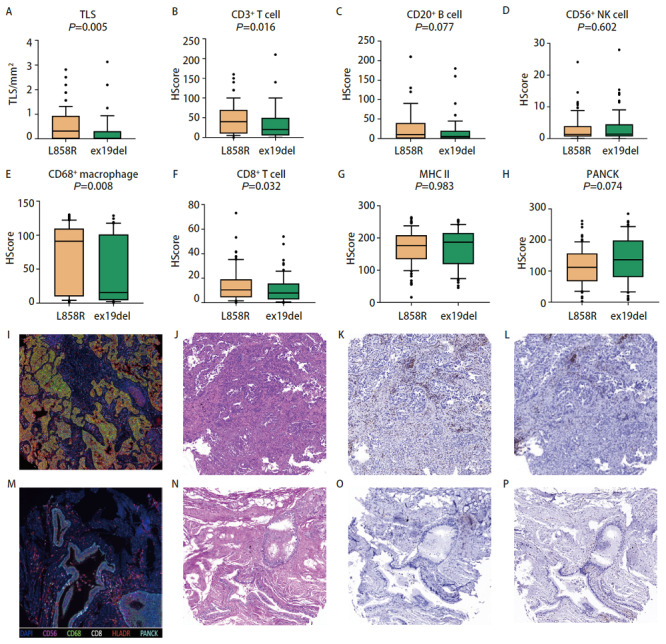
EGFR敏感突变亚型的免疫浸润差异分析。 A-H：EGFR外显子21 L858R突变与EGFR外显子19缺失突变的肺腺癌组织中各类免疫细胞浸润以及TLS形成情况；I-L：典型的EGFR外显子21 L858R的肺腺癌mIHC图像、HE染色图像以及IHC CD3和IHC CD20图像（×100）；M-P：典型的EGFR外显子19缺失突变的肺腺癌mIHC图像、HE染色图像以及IHC CD3和IHC CD20图像（×100）。

### 2.6 EGFR常见敏感突变亚型与免疫细胞分布的空间异质性分析

我们进一步比较了EGFR常见的两种突变亚型外显子21 L858R突变与外显子19缺失突变之间免疫细胞浸润的空间分布差异，我们发现EGFR外显子21 L858R突变患者的肿瘤组织内CD3^+ ^T细胞（P=0.02）浸润的空间异质性更高，CD56^+^自然杀伤细胞（P=0.085）、CD68^+^巨噬细胞（P=0.052）以及CD8^+ ^T细胞（P=0.19）有空间异质性更高的趋势，而TLS分布情况以及CD20^+ ^B细胞、MHC II类抗原呈递细胞等免疫细胞空间分布情况在两种突变亚型之间的差异并不显著（图6）。整体而言，EGFR外显子21 L858R突变的肿瘤组织中，免疫细胞的空间分布异质性较大。

**图6:**
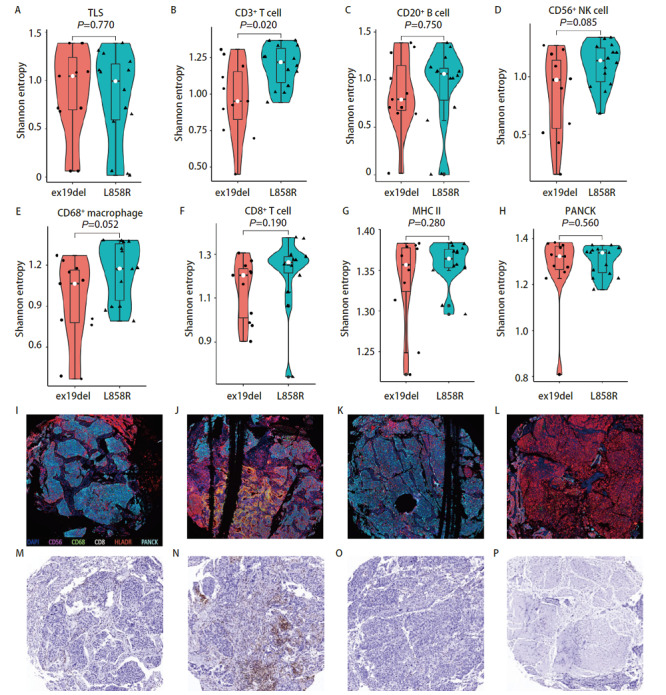
EGFR敏感突变亚型的免疫细胞分布的空间异质性分析。 A-H：EGFR外显子21 L858R突变与EGFR外显子19缺失突变的肺腺癌组织中各类免疫细胞以及TLS的空间分布差异；I-L：典型的EGFR外显子21 L858R突变的肺腺癌同一肿瘤内部不同采样位点的mIHC图像（×100）；M-P：典型的EGFR外显子21 L858R突变的肺腺癌同一肿瘤内部不同采样位点的IHC CD3图像（×100）。

### 2.7 免疫细胞浸润与EGFR突变患者预后的相关性分析

为了探索不同免疫细胞浸润的情况与EGFR突变患者预后的关系，我们以各类免疫细胞浸润的中值将患者分为免疫细胞浸润高低两组，分析其与无病生存期（disease free survival, DFS）之间的相关性。我们发现EGFR突变肺腺癌患者中，TLS形成数量多（P=0.0079）、CD3^+ ^T细胞浸润数量多（P=0.0135）以及CD20^+ ^B细胞浸润数量多（P=0.0451）提示更好的预后。而CD56^+^自然杀伤细胞（P=0.0960）、CD68^+^巨噬细胞（P=0.6729）、CD8^+ ^T细胞（P=0.1403）、MHC II类抗原呈递细胞（P=0.0963）的浸润数量与预后的关系均无统计学差异（图7）。

**图7:**
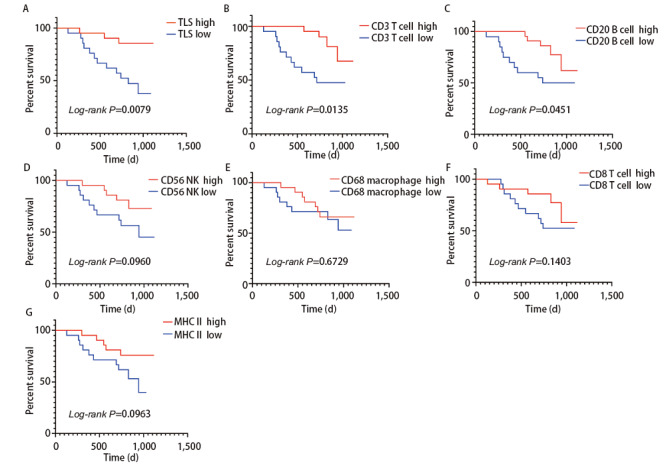
EGFR突变肺腺癌患者中各类免疫细胞及TLS数量与预后之间的关系。 A：TLS数量与EGFR突变肺腺癌患者预后之间的关系；B：CD3^+ ^T细胞数量与EGFR突变肺腺癌患者预后之间的关系；C：CD20^+ ^B细胞数量与EGFR突变肺腺癌患者预后之间的关系；D：CD56^+^自然杀伤细胞数量EGFR突变肺腺癌患者预后之间的关系；E：CD68^+^巨噬细胞数量与EGFR突变肺腺癌患者预后之间的关系；F：CD8^+ ^T细胞数量与EGFR突变肺腺癌患者预后之间的关系；G：MHC II类细胞数量与EGFR突变肺腺癌患者预后之间的关系。

### 2.8 免疫细胞分布的空间异质性与EGFR突变患者预后的相关性分析

为了探索各类免疫细胞空间分布的情况与EGFR突变患者预后的关系，我们以肿瘤组织内各类免疫细胞浸润的Shannon entropy的中位数将患者分为免疫细胞空间分布异质性高低两组，并分析其与DFS之间的相关性。我们发现EGFR突变肺腺癌患者中，CD8^+ ^T细胞空间分布异质性高与更好的预后相关（P=0.0315）。而TLS、CD3^+ ^T细胞、CD20^+ ^B细胞、CD56^+^自然杀伤细胞、CD68^+^巨噬细胞以及MHC II类抗原呈递细胞的空间分布情况与预后无明显的相关性（图8）。

**图8:**
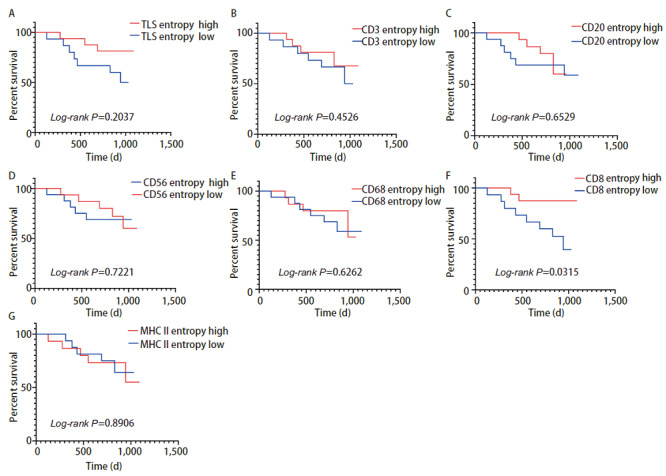
EGFR突变肺腺癌患者中各类免疫细胞及TLS空间分布与预后之间的关系。 A：TLS空间分布与EGFR突变肺腺癌患者预后之间的关系；B：CD3^+ ^T细胞空间分布与EGFR突变肺腺癌患者预后之间的关系；C: CD20^+ ^B细胞空间分布与EGFR突变肺腺癌患者预后之间的关系；D：CD56^+^自然杀伤细胞空间分布与EGFR突变肺腺癌患者预后之间的关系；E：CD68^+^巨噬细胞空间分布与EGFR突变肺腺癌患者预后之间的关系；F：CD8^+ ^T细胞空间分布与EGFR突变肺腺癌患者预后之间的关系；G：MHC II类细胞空间分布与EGFR突变肺腺癌患者预后之间的关系。

## 3 讨论

目前，肺癌的发病率及死亡率均位居各类恶性肿瘤的前列，其中最常见的组织学类型是肺腺癌，长期以来治疗手段以手术、放疗、化疗为主，近年来EGFR-TKIs靶向治疗以及免疫治疗为肺腺癌患者带来了新的希望^[16]^。EGFR突变肺腺癌患者大多可以从EGFR-TKIs治疗中获益，但是似乎对免疫治疗获益不佳，即使PD-L1表达高的患者也没有从免疫治疗中获得更好的获益，这可能与其独特的免疫微环境有关^[17]^。一项基于单细胞层面的研究^[18]^表明EGFR突变肺腺癌患者的肿瘤免疫微环境受到抑制，CD8^+^组织驻留记忆性T细胞（tissue resident memory T cell, TRM）减少，TLS形成数量更少，并可通过分泌细胞因子招募各种免疫抑制细胞，免疫检查点表达更低，这种独特的抑制性免疫微环境可能是免疫治疗效果不佳的原因。另一项基于癌症基因组图谱（The Cancer Genome Atlas, TCGA）公共数据集的研究^[19]^表明，EGFR突变阳性的肺腺癌患者的TME中缺少记忆B细胞、浆细胞、CD8^+ ^T细胞等免疫细胞的浸润，而且基因富集分析发现EGFR突变患者与自然杀伤细胞有关的三条通路：自然杀伤细胞介导的对肿瘤细胞的免疫应答的正调控、自然杀伤细胞激活参与免疫反应以及自然杀伤细胞介导的对肿瘤细胞的免疫应答均处于下调的状态。本研究主要通过IHC以及mIHC的技术从蛋白层面对EGFR突变的肺腺癌患者TME中免疫细胞浸润情况进行探讨。此外，通过比较肿瘤组织不同采样部位之间的免疫细胞浸润的差异，对肿瘤内免疫细胞分布的空间异质性进行了探索。

我们发现了EGFR突变肺腺癌患者TME中CD56^+^自然杀伤细胞以及CD8^+ ^T细胞浸润的较少，而CD68^+^巨噬细胞以及MHC II类抗原呈递细胞浸润更多，并且通过mIHC染色图可以直观地观察到EGFR突变与野生型之间免疫细胞浸润的景观差异。EGFR突变与野生型肺腺癌之间免疫细胞分布的总体空间差异不大，我们发现EGFR野生型的肺腺癌组织中MHC II类抗原呈递细胞的空间分布异质性较大。我们进一步比较了EGFR VAF与免疫细胞浸润的相关性，结果表明EGFR VAF高的肿瘤组织中免疫细胞是低浸润状态，包括CD3^+ ^T细胞、CD20^+ ^B细胞、CD56^+^自然杀伤细胞、CD68^+^巨噬细胞、CD8^+ ^T细胞等多种免疫细胞浸润数量都是减少的，而且TLS形成的数量更少，PANCK标记的肿瘤细胞含量更多。而在EGFR VAF低的肿瘤组织中，虽然CD3^+ ^T细胞的浸润相对较高，但是在肿瘤内的空间分布上却呈现出较大的异质性。一项临床研究^[20]^表明接受EGFR-TKIs治疗的肺腺癌患者中，EGFR VAF高的患者相比EGFR VAF低的患者可以获得更长的无进展生存期和总生存期，我们发现EGFR VAF低的肿瘤组织有着更高的免疫细胞浸润，也是表明了EGFR VAF低的患者如果EGFR-TKIs治疗效果不佳，或许可以从免疫治疗中寻求更多获益。EGFR突变最常见的两种突变亚型为外显子19缺失突变与外显子21 L858R突变，两者都是EGFR-TKIs治疗的敏感突变，但是部分研究^[12]^发现外显子19缺失突变对阿法替尼、埃克替尼等TKIs药物获益更多，而部分研究^[12]^表明外显子21 L858R突变患者对免疫治疗获益更多，我们的研究结果表明，外显子19缺失突变的肺腺癌肿瘤组织中CD3^+ ^T细胞、CD20^+ ^B细胞、CD68^+^巨噬细胞、CD8^+ ^T细胞等免疫细胞浸润更少，TLS形成的数量也更少。外显子21 L858R突变患者的肿瘤组织中虽然相较于外显子19缺失突变的肿瘤组织有较多的免疫细胞浸润，但是包括CD3^+ ^T细胞、CD8^+ ^T细胞、CD56^+^自然杀伤细胞以及CD68^+^巨噬细胞在内的多种免疫细胞都表现出更高的空间分布的异质性。预后分析发现TLS形成的数量多，CD3^+ ^T细胞以及CD20^+ ^B细胞浸润高与EGFR突变肺腺癌患者更好的DFS有关，而且CD8^+ ^T细胞空间分布异质性高与更好的预后相关。

本研究通过IHC以及mIHC对肺腺癌患者中EGFR突变状态与免疫微环境特征的相关性进行了分析，我们发现了EGFR突变状态与免疫细胞的浸润数量以及空间分布有潜在的联系，具体的机制需要进一步的研究来证实。本研究的样本量较少，研究结果可能会存在一定偏差，而且研究的免疫细胞种类较少，更多的免疫细胞亚群特征仍需进一步的研究来探索。
